# Saliva of *Therioaphis trifolii* (Drepanosiphidae) Activates the SA Plant Hormone Pathway, Inhibits the JA Plant Hormone Pathway, and Improves Aphid Survival Rate

**DOI:** 10.3390/ijms252312488

**Published:** 2024-11-21

**Authors:** Kaihui Zhu, Neng Zhang, Daogang Zhang, Ni Cai, Rong Liu, Hui Dong, Zehua Zhang, Xiongbing Tu

**Affiliations:** 1 State Key Laboratory for Biology of Plant Diseases and Insect Pests, Institute of Plant Protection, Chinese Academy of Agricultural Sciences, Beijing 100193, China; zkh0.0@hotmail.com (K.Z.); zhang1998neng@163.com (N.Z.); ippleslie04@163.com (D.Z.); 15027173107@163.com (N.C.); liurong20220622@163.com (R.L.); zhangzehua@caas.cn (Z.Z.); 2 Key Laboratory of Economical and Applied Entomology of Liaoning Province, College of Plant Protection, Shenyang Agriculture University, Shenyang 110866, China

**Keywords:** *Medicago sativa*, *Therioaphis trifolii*, saliva, SA plant hormone pathway, JA plant hormone pathway

## Abstract

The spotted alfalfa aphid (*Therioaphis trifolii*) is a kind of destructive pest of cultivated alfalfa (*Medicago sativa*) that results in significant financial losses for the livestock sector. To understand how *T. trifolii* navigates the biochemical defenses of its host, we investigated the effects of susceptible and resistant aphid strains on two alfalfa cultivars. *T. trifolii* was reared for over 50 generations on two cultivars—WL343, which is susceptible to *T. trifolii*, and Zhongmu No. 1, which is resistant—resulting in the development of a resistant aphid strain (R-aphid) and a susceptible aphid strain (S-aphid). The results showed that the survival rate of R-aphids was significantly higher than that of S-aphids (*p* = 0.039) on the resistant cultivar Zhongmu No. 1, while there was no significant difference in survival rates between the two aphid strains on WL343 (*p* = 0.139). This suggests that S- and R-aphids differ in their ability to modulate plant defense mechanisms, influencing their survival rates. The application of saliva from R-aphids reared on Zhongmu No. 1 (R-saliva) reduced the repellency and toxicity of treated plants, improving aphid survival. Furthermore, R-aphid infestation and R-saliva application activated the salicylic acid (SA) signaling pathway while suppressing the jasmonic acid (JA) pathway, enhancing the host plant’s suitability for aphid colonization. We propose that R-aphids may use their saliva to activate the SA pathway, which in turn inhibits JA synthesis, weakening the plant’s defenses. These findings provide valuable insights into how *T. trifolii* interacts with host plant defense systems.

## 1. Introduction

In the natural environment, plants are susceptible to herbivory by insects; consequently, they have developed a range of defensive mechanisms to mitigate the impact of pest infestations [1]. Conversely, insects have evolved proactive strategies to counteract the defensive mechanisms employed by plants [2]. Consequently, plants and insects have established a complex interplay of defensive and counter-defensive mechanisms as a result of coevolution [3,4]. For instance, plants synthesize phenolic compounds and flavonoids as a defensive response to insect herbivory, thereby inhibiting the pests’ capacity to assimilate nutrients and develop [5]. Insects possess the ability to adapt to plant defense mechanisms by synthesizing detoxifying enzymes, which facilitate their survival and development. Notable examples of these enzymes include carboxylesterases and cathepsins [6].

The initial phase in the establishment of inducible insect resistance in plants involves the identification of associated pattern molecules, referred to as herbivore-associated molecular patterns (HAMPs), which are specific elicitors for insects. Following the activation of defense signaling pathways, there is an upregulation in the expression of defense-related genes. The accumulation of defensive compounds and proteins will ultimately enhance plant resistance to insect infestations, thereby impeding the proliferation of these pests [7]. As noted by Ton, the signaling pathways associated with salicylic acid (SA) and jasmonic acid (JA) plant hormones predominantly play a crucial role in the activation of inducible defenses in plants [7]. For instance, the enhancement of insect resistance in plants can be attributed to the upregulation of two critical genes within the jasmonic acid (JA) plant hormone signaling pathway, namely AOS and LOX [8]. Vadassery demonstrated that an elevation in the signaling of the JA plant hormone within the roots of Nicotiana tabacum resulted in a significant increase in the overall folate content of the plant, which in turn substantially decreased the survival rate of *Spodoptera litoralis* (Boisd) [9].

Research from Fan demonstrated that the application of exogenous jasmonic acid (JA) to *Chrysanthemum morifolium* resulted in a notable decrease in the survival rate of *Macrosiphoniella sanborni* [10]. *ICS* and *PAL* are critical genes within the salicylic acid (SA) signaling pathway in plants. Furthermore, as a component of the pathogen signaling network, salicylic acid plays a vital role in facilitating indirect resistance to insect herbivory in plant systems [11]. Remco (2002) reported that when *Arabidopsis thaliana* was infected with *Pieris rapae*, it emitted methyl salicylate, a byproduct of the JA signal pathway that is attractive to the predator *Apanteles ruficrus* [12]. Furthermore, Ozawa indicated that the infection of *Phaseolus lunatus* by *Tetranychus cinnabarinus* results in the release of volatile compounds that are activated by the salicylic acid (SA) plant hormone signaling pathway. These volatile chemicals serve to attract the natural predators of *T. cinnabarinus*, including *Scolothrips sexmaculatus* [13].

Insects have evolved a range of counter-defensive strategies in response to the defensive mechanisms employed by plants, and this is essential for their population sustainability [14]. *Spodoptera litura* can mitigate the toxicity of plant protease inhibitors by enhancing the production of tissue protein-cysteine-digesting enzymes [15]. The activity of detoxification enzymes present in saliva, including carboxylesterase, significantly escalated when *Heliothis assulta* was nourished with its host plant, thereby serving to counteract the defensive toxins produced by the plant [16]. Saliva functions as a crucial intermediary between phytophagous insects and plants within their mutual counter-defensive strategies. It comprises both effectors, which can impede plant defense mechanisms and enhance the survival of the insects, as well as elicitors, which can activate plant defense responses [17].

Ji illustrated the role of the detoxifying enzyme β-1,4-glucanase (*NIEG1*) present in the saliva of *Nilaparvata lugens* in facilitating the feeding process of *N. lugens* on *Oryza sativa*. This enzyme plays a significant role in the breakdown of cellulose present in the cell walls of plants [18]. Through its interaction with jasmonic acid (JA)-related receptors, *NIEG1* is capable of concurrently inhibiting the plant’s defensive response that is mediated by JA. Additionally, the plant immune regulatory protein WRKY33 engages directly with the effector protein BSP-9, which is present in the saliva of the whitefly *Bemisia tabaci*, thereby attenuating the immunological signaling of plants [19]. Consequently, the complex interplay between insect herbivory, plant defense mechanisms, and insect counter-defenses is significantly influenced by the composition of insect saliva. Throughout the evolutionary process, insects have persistently adapted the constituents of their saliva in response to plant defense strategies.

This study examined *T. trifolii* on two *M. sativa* cultivars: aphid-susceptible WL343 and aphid-resistant Zhongmu No. 1. Over 50 generations, susceptible (S-aphid) and resistant (R-aphid) strains emerged. Survival and hormonal responses revealed differences, suggesting distinct salivary impacts on plant defenses, aiding aphid management insights.

## 2. Results

### 2.1. The Survival Rate of R-Aphid on Aphid-Resistant Alfalfa (Zhongmu No. 1) Was Significantly Higher Than That of S-Aphid

**Figure 1:**
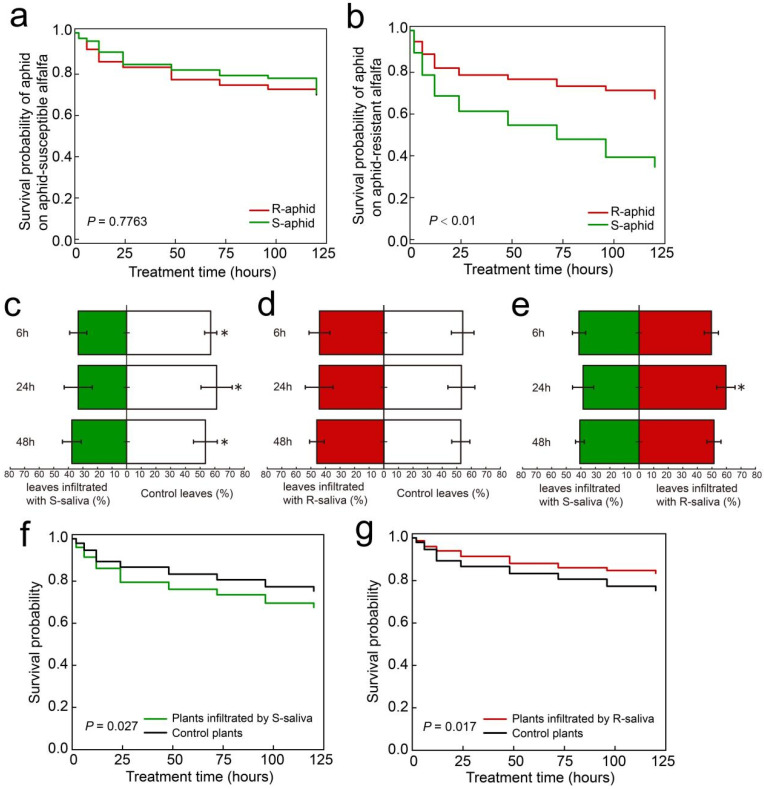
Mortality of aphids previously reared on aphid susceptible cv. WL343 or resistant cv. Zhongmu No. 1 alfalfa and transferred to either cv. WL343 (**a**) or cv. Zhongmu No. 1. (**b**), with mortality measured over a 120 h period. Behavioral selection by aphids provided with leaves treated with saliva collected from either the aphid susceptible cv. WL343 (**c**), or the aphid resistant cv. Zhongmu No. 1 (**d**) compared to the control leaves or between the two saliva treatments (**e**) at 6, 24, and 48 h. (**f**,**g**) Mortality of naive aphids infecting leaves after either the S-saliva or R-saliva infiltration treatment compared to control plants. The “R-aphid” and “S-aphid” in A and B represent *T. trifolii* reared on aphid-resistant alfalfa (Zhongmu No. 1) and susceptible alfalfa (WL343) for more than 50 generations, respectively. The “*” in (**a**,**b**,**f**,**g**) indicate significant differences in mortality (*p* < 0.05) using the log rank (Kaplan–Cox) test. The “*” in (**c**,**d**,**e**) indicate significant differences in aphid numbers (*p* < 0.05) using Tukey’s HSD test.

The mortality risk between R-aphids and S-aphids when they infected Zhongmu No. 1 was significantly different (χ^2^ = 30.678, DF = 1, *p* < 0.0001). The mortality risk of S-aphid was 2.5589 times that of R-aphid, which was significantly higher than that of R-aphid (z = 5.35, *p* < 0.0001) (Figure 1a), but when R-aphids and S-aphids were infecting aphid-susceptible alfalfa (WL343), the mortality risk between R-aphids and S-aphids had no significant difference (χ^2^ = 0.0808, DF = 1, *p* = 0.7763) (Figure 1b). Therefore, Zhongmu No. 1 was selected as the host plant for follow-up evaluation.

### 2.2. Aphids Preferred Leaves Treated with R-Saliva

The behavioral selection experiment demonstrated that after 6, 24, and 48 h, there were considerably more aphids remaining on the control leaves than on the leaves where S-saliva had been infiltrated (6 h, z = 12.32, *p* = 0.038; 24 h, z = 1.32, *p* = 0.024; 48 h, z = 11.65, *p* = 0.036) (Figure 1c). However, there was no significant difference in the number of aphids that remained on the control leaves and those that remained on the leaves with R-saliva infiltration after 6, 24, and 48 h (Figure 1d). After 24 h, the quantity of aphids on leaves infiltrated with R-saliva was considerably greater than on the leaves infiltrated with S-saliva (24 h, z = 8.65, *p* = 0.034) (Figure 1e).

### 2.3. Effect of Saliva Infiltration on Aphid Survival Rate

A naive aphid cohort fed an artificial diet for 7 days was exposed to leaves treated with either R- or S-saliva. The results showed that at 120 h, there was a significant difference between the mortality risks of the aphids feeding on leaves with S-saliva infiltration and the aphids feeding on leaves with the control treatment (χ^2^ = 4.8907, DF = 1, *p* = 0.027) (Figure 1f).

The mortality risks of the aphids feeding on leaves with R-saliva infiltration and aphids feeding on leaves with the control treatment were significantly different at 120 h (χ^2^ = 5.6625, DF = 1, *p* = 0.017) (Figure 1g).

### 2.4. R-Saliva Activates the Salicylic Acid Synthesis Signal Pathway in Alfalfa

To verify the difference in the ability of R-aphids and S-aphids inducing defense responses in alfalfa, we measured the changes in the content of jasmonic acid and salicylic acid in the leaves of WL343 cultivar alfalfa and Zhongmu No. 1 cultivar alfalfa as well as the expression levels of key synthetic genes after being infected by two strains of *T. trifolii* (R-aphid and S-aphid).

**Figure 2:**
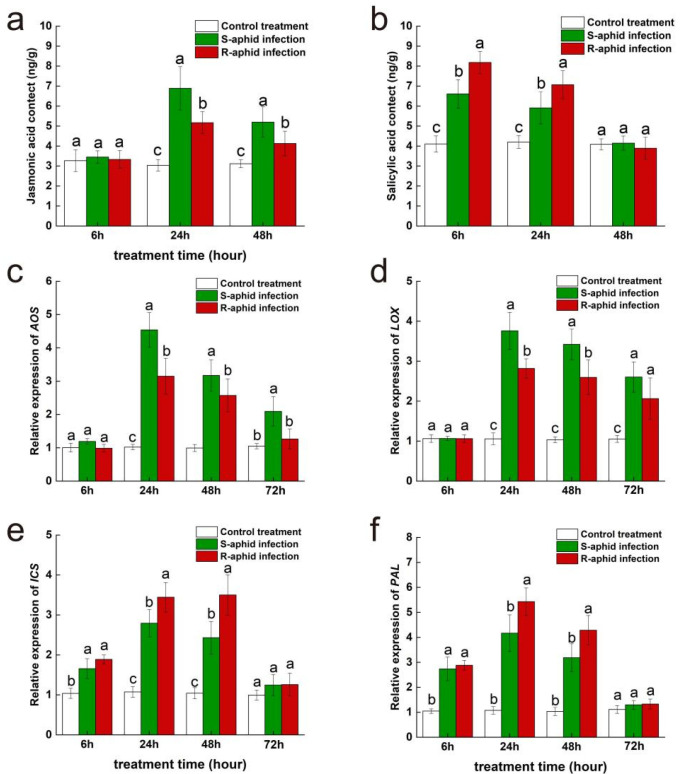
The effect of Zhongmu No. 1 cultivar alfalfa infested by either R-aphids or S-aphids on the subsequent activities of the JA plant hormone (**a**) and SA plant hormone (**b**) signaling pathways and expression of the *AOS* gene (**c**), the *LOX* gene (**d**), the *ICS* gene (**e**), and the *PAL* gene (**f**). The different lowercase letters indicate significant differences in signal hormone contents and gene expression between Zhongmu No. 1 infested by R-aphids and S-aphids (*p* < 0.05; Tukey’s HSD).

At the 24th and 48th hours after being infected by *T. trifolii*, jasmonic acid content of Zhongmu No. 1 was significantly influenced by the strain of *T. trifolii* (24 h: F_2,14_ = 28.44, *p* < 0.05; 48 h: F_2,14_ = 17.03, *p* < 0.05) (Figure 2a). However, the 6th hour after being infected by two strains of *T. trifolii*, jasmonic acid content of Zhongmu No. 1 had no significant difference from the blank control. Additionally, at the 24th and 48th hours after being infected by *T. trifolii*, the jasmonic acid content of alfalfa infected by S-aphids (24 h, 6.89 ± 1.08 mg/g; 48 h, 5.20 ± 0.76 mg/g) was significantly higher than of that infected by R-aphids (24 h, 5.17 ± 0.55 mg/g; 48 h, 3.76 ± 0.62 mg/g) (24 h, z = 1.94, *p* < 0.05; 48 h, z = 2.08, *p* < 0.05), and they were both significantly higher than the blank control (24 h, 3.03 ± 0.29 mg/g; 48 h, 3.11 ± 0.20 mg/g) (24 h, S-aphid-infected vs. control treatment, z = 6.23, *p* < 0.05; 24 h, R-aphid-infected vs. control treatment, z = 12.52, *p* < 0.05; 48 h, S-aphid-infected vs. control treatment, z = 9.52, *p* < 0.05; 48 h, R-aphid-infected vs. control treatment, z = 8.52, *p* < 0.05).

At the 6th and 24th hours after being infected by *T. trifolii*, salicylic acid content of Zhongmu No. 1 was significantly influenced by the strain of *T. trifolii* (6 h: F_2,14_ = 61.91, *p* < 0.05; 24 h: F_2,14_ = 29.87, *p* < 0.05) (Figure 2b). Besides, at the 6th and 24th hours after being infected by *T. trifolii*, salicylic acid content of alfalfa infected by R-aphids (6 h, 8.18 ± 0.55 mg/g; 24 h, 7.07 ± 0.70 mg/g) was significantly higher than that infected by S-aphids (6 h, 6.61 ± 0.71 mg/g; 24 h, 5.91 ± 0.80 mg/g) (6 h, z = 11.76, *p* < 0.05; 24 h, z = 6.64, *p* < 0.05), and they both were significantly higher than the blank control (6 h, 4.10 ± 0.41 mg/g; 24 h, 4.20 ± 0.32 mg/g) (6 h, S-aphid-infected vs. control treatment, z = 5.32, *p* < 0.05; 6 h, R-aphid-infected vs. control treatment, z = 11.20, *p* < 0.05; 24 h, S-aphid-infected vs. control treatment, z = 5.94, *p* < 0.05; 24 h, R-aphid-infected vs. control treatment, z = 16.54, *p* < 0.05). However, at the 48th hour after being infected by two strains of *T. trifolii*, the salicylic acid content of Zhongmu No. 1 had returned to a level which was of no significant difference compared to the blank control.

At the 24th and 48th hours after being infected by *T. trifolii*, expression level of *AOS*, a key synthetic gene of jasmonic acid was significantly influenced by the strain of *T. trifolii* (24 h: F_2,14_ = 97.02, *p* < 0.05; 48 h: F_2,14_ = 44.62, *p* < 0.05) (Figure 2c). However, at the 6th hour after being infected by two strains of *T. trifolii*, the *AOS* expression level of Zhongmu No. 1 had no significant difference from the blank control. Additionally, at the 24th and 48th hours after being infected by *T. trifolii*, the *AOS* expression level of Zhongmu No. 1 infected by S-aphids (24 h, 4.54 ± 0.52; 48 h, 3.17 ± 0.46) was significantly higher than of that infected by R-aphids (24 h, 3.15 ± 0.54; 48 h, 2.57 ± 0.50) (24 h, z = 11.24, *p* < 0.05; 48 h, z = 7.54, *p* < 0.05), and they were both significantly higher than the blank control (24 h, 1.02 ± 0.08; 48 h, 0.99 ± 0.10) (24 h, S-aphid-infected vs. control treatment, z = 12.95, *p* < 0.05; 24 h, R-aphid-infected vs. control treatment, z = 16.24, *p* < 0.05; 48 h, S-aphid-infected vs. control treatment, z = 11.25, *p* < 0.05; 48 h, R-aphid-infected vs. control treatment, z = 12.54, *p* < 0.05). At the 72nd hour after being infected by *T. trifolii*, there was no significant difference in expression levels of *AOS* between Zhongmu No. 1 infected by R-aphids (72 h, 1.48 ± 0.30) and S-aphids (72 h, 2.09 ± 0.44), but they were both significantly higher than the blank control (72 h, 1.05 ± 0.08) (72 h, R-aphid-infected vs. control treatment, z = 13.54, *p* < 0.05; 72 h, S-aphid-infected vs. control treatment, z = 12.65, *p* < 0.05).

At the 24th and 48th hour after being infected by *T. trifolii*, expression levels of *LOX*, a key synthetic gene of jasmonic acid, was significantly influenced by the strain of *T. trifolii* (24 h: F_2,14_ = 94.17, *p* < 0.05; 48 h: F_2,14_ = 55.87, *p* < 0.05) (Figure 2d). However, at the 6th hour after being infected by two strains of *T. trifolii*, the *LOX* expression level of Zhongmu No. 1 had no significant difference from the blank control. Additionally, at the 24th and 48th hours after being infected by *T. trifolii*, the *LOX* expression level of Zhongmu No. 1 infected by S-aphids (24 h, 3.76 ± 0.46; 48 h, 3.42 ± 0.38) was significantly higher than of that infected by R-aphids (24 h, 2.82 ± 0.24; 48 h, 3.00 ± 0.43) (24 h, z = 16.85, *p* < 0.05; 48 h, z = 8.64, *p* < 0.05), and they were both significantly higher than the blank control (24 h, 1.06 ± 0.15; 48 h, 1.03 ± 0.07) (24 h, S-aphid-infected vs. control treatment, z = 12.54, *p* < 0.05; 24 h, R-aphid-infected vs. control treatment, z = 10.57, *p* < 0.05; 48 h, S-aphid-infected vs. control treatment, z = 16.78, *p* < 0.05; 48 h, R-aphid-infected vs. control treatment, z = 13.57, *p* < 0.05). At the 72nd hour after being infected by *T. trifolii*, there was no significant difference in expression level of *LOX* between Zhongmu No. 1 infected by R-aphids (72 h, 2.60 ± 0.38) and S-aphids (72 h, 2.06 ± 0.52), but they were both significantly higher than the blank control (72 h, 1.06 ± 0.09) (72 h, R-aphid-infected vs. control treatment, z = 13.89, *p* < 0.05; 72 h, S-aphid-infected vs. control treatment, z = 19.85, *p* < 0.05).

At the 24th and 48th hours after being infected by *T. trifolii*, expression level of *ICS*, a key synthetic gene of salicylic acid was significantly influenced by the strain of *T. trifolii* (24 h: F_2,14_ = 53.64, *p* < 0.05; 48 h: F_2,14_ = 51.62, *p* < 0.05) (Figure 2e). At the 6th hour after being infected by two strains of *T. trifolii*, there was no significant difference in expression level of ICS between Zhongmu No. 1 infected by R-aphids (6 h, 1.66 ± 0.25) and S-aphids (6 h, 1.89 ± 0.11), but they were both significantly higher than the blank control (6 h, 1.04 ± 0.13) (6 h, R-aphid-infected vs. control treatment, z = 10.94, *p* < 0.05; 6 h, S-aphid-infected vs. control treatment, z = 13.84, *p* < 0.05). At the 24th and 48th hours after being infected by *T. trifolii*, the *ICS* expression level of Zhongmu No. 1 infected by R-aphids (24 h, 3.44 ± 0.37; 48 h, 3.00 ± 0.51) was significantly higher than that infected by S-aphids (24 h, 2.79 ± 0.34; 48 h, 2.43 ± 0.41) (24 h, z = 11.24, *p* < 0.05; 48 h, z = 5.84, *p* < 0.05), and they were both significantly higher than the blank control (24 h, 1.07 ± 0.13; 48 h, 1.04 ± 0.14) (24 h, S-aphid-infected vs. control treatment, z = 9.51, *p* < 0.05; 24 h, R-aphid-infected vs. control treatment, z = 6.54, *p* < 0.05; 48 h, S-aphid-infected vs. control treatment, z = 11.27, *p* < 0.05; 48 h, R-aphid-infected vs. control treatment, z = 9.65, *p* < 0.05). However, at the 72nd hour after being infected by *T. trifolii*, only the *ICS* expression level of Zhongmu No. 1 infected by R-aphids (72 h, 0.99 ± 0.13) was significantly higher than the blank control (72 h, R-aphid-infected vs. control treatment, z = 8.64, *p* < 0.05), *ICS* expression level of Zhongmu No. 1 infected by S-aphids had returned to a level which was of no significant difference compared to the blank control.

At the 24th and 48th hours after being infected by *T. trifolii*, expression level of *PAL*, a key synthetic gene of salicylic acid was significantly influenced by the strain of *T. trifolii* (24 h: F_2,14_ = 26.70, *p* < 0.05; 48 h: F_2,14_ = 14.63, *p* < 0.05) (Figure 2f). At the 6th hour after being infected by two strains of *T. trifolii*, there was no significant difference in expression levels of PAL between Zhongmu No. 1 infected by R-aphids (6 h, 2.88 ± 0.19) and S-aphids (6 h, 2.74 ± 0.47), but they were both significantly higher than the blank control (6 h, 1.04 ± 0.16) (6 h, R-aphid-infected vs. control treatment, z = 8.54, *p* < 0.05; 6 h, S-aphid-infected vs. control treatment, z = 7.51, *p* < 0.05). At the 24th and 48th hours after being infected by *T. trifolii*, *PAL* expression level of Zhongmu No. 1 infected by R-aphids (24 h, 5.43 ± 0.55; 48 h, 4.28 ± 0.59) was significantly higher than of that infected by S-aphids (24 h, 4.17 ± 0.73; 48 h, 3.18 ± 0.56) (24 h, z = 11.95, *p* < 0.05; 48 h, z = 16.85, *p* < 0.05), and they were both significantly higher than the blank control (24 h, 1.08 ± 0.15; 48 h, 1.03 ± 0.16) (24 h, S-aphid-infected vs. control treatment, z = 9.51, *p* < 0.05; 24 h, R-aphid-infected vs. control treatment, z = 9.54, *p* < 0.05; 48 h, S-aphid-infected vs. control treatment, z = 11.27, *p* < 0.05; 48 h, R-aphid-infected vs. control treatment, z = 4.56, *p* < 0.05). However, at the 72nd hour after being infected by two strains of *T. trifolii*, the *PAL* expression level of Zhongmu No. 1 returned to a level which was of no significant difference compared to the blank control.

**Figure 3:**
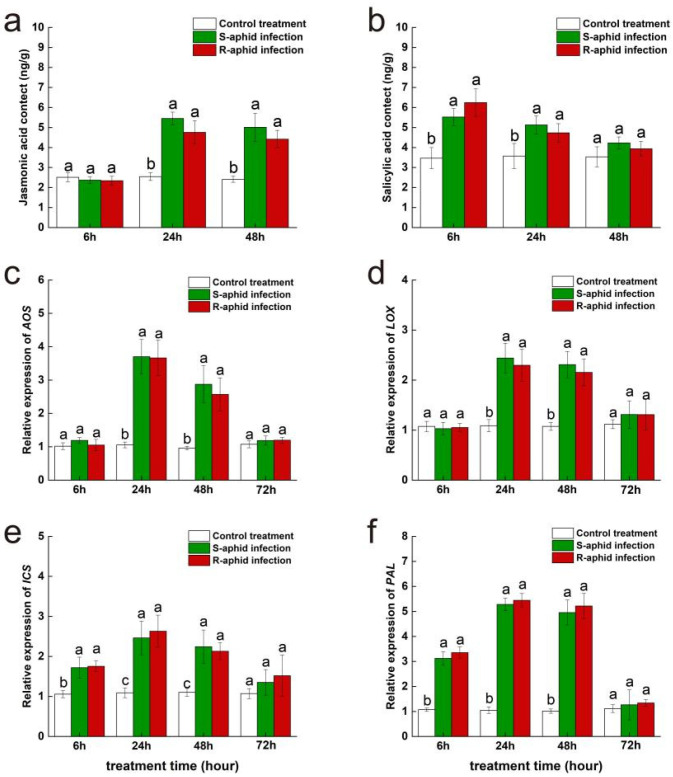
The effect of WL343 cultivar alfalfa infested by either R-aphids or S-aphids on the subsequent activities of the JA plant hormone (**a**) and SA plant hormone (**b**) signaling pathways and expression of the *AOS* gene (**c**), the *LOX* gene (**d**), the *ICS* gene (**e**), and the *PAL* gene (**f**). The different lowercase letters indicate significant differences in signal hormone contents and gene expression between Zhongmu No. 1 infested by R-aphids and S-aphids (*p* < 0.05; Tukey’s HSD).

At the 6th, 24th, and 48th hours after being infected by *T. trifolii*, there was no significant difference in jasmonic acid content between WL343 leaves infected by S-aphids and R-aphids (Figure 3a). At the 6th hour after being infected by two strains of *T. trifolii*, jasmonic acid content of Wl343 had no significant difference compared to the blank control. At the 24th and 48th hours after being infected by *T. trifolii*, the jasmonic acid contents of WL343 infected by S-aphids (24 h, 5.45 ± 0.32 mg/g; 48 h, 5.01 ± 0.70 mg/g) and R-aphids (24 h, 3.70 ± 0.59 mg/g; 48 h, 4.42 ± 0.44 mg/g) were both significantly higher than the blank control (24 h, 2.54 ± 0.20 mg/g; 48 h, 2.41 ± 0.15 mg/g) (24 h, S-aphid-infected vs. control treatment, z = 7.52, *p* < 0.05; 24 h, R-aphid-infected vs. control treatment, z = 5.94, *p* < 0.05; 48 h, S-aphid-infected vs. control treatment, z = 12.84, *p* < 0.05; 48 h, R-aphid-infected vs. control treatment, z = 10.54, *p* < 0.05).

At the 6th, 24th, and 48th hours after being infected by *T. trifolii*, there was no significant difference in salicylic acid content between WL343 leaves infected by S-aphids and R-aphids (Figure 3b). At the 6th and 24th hours after being infected by *T. trifolii*, the salicylic acid contents of WL343 infected by S-aphids (6 h, 5.52 ± 0.42 mg/g; 24 h, 5.13 ± 0.45 mg/g) and R-aphid (6 h, 6.25 ± 0.68 mg/g; 24 h, 4.73 ± 0.46 mg/g) were both significantly higher than the blank control (6 h, 3.47 ± 0.53 mg/g; 24 h, 3.57 ± 0.62 mg/g) (6 h, S-aphid-infected vs. control treatment, z = 11.95, *p* < 0.05; 6 h, R-aphid-infected vs. control treatment, z = 16.25, *p* < 0.05; 24 h, S-aphid-infected vs. control treatment, z = 13.54, *p* < 0.05; 24 h, R-aphid-infected vs. control treatment, z = 16.52, *p* < 0.05). However, at the 48th hour after being infected by two strains of *T. trifolii*, the salicylic acid content of WL343 had returned to a level which was of no significant difference compared to the blank control.

At the 6th, 24th, and 48th hours after being infected by *T. trifolii*, there was no significant difference in the expression levels of *AOS* (a key synthetic gene of jasmonic acid) of WL343 leaves infected by S-aphids and R-aphids (Figure 3c). At the 6th hour after being infected by two strains of *T. trifolii*, the *AOS* expression level of Wl343 had no significant difference from the blank control. At the 24th and 48th hours after being infected by *T. trifolii*, the *AOS* expression levels of WL343 infected by S-aphids (24 h, 3.70 ± 0.52; 48 h, 2.87 ± 0.55) and R-aphids (24 h, 3.70 ± 0.59; 48 h, 4.42 ± 0.44) were both significantly higher than the blank control (24 h, 3.67 ± 0.53; 48 h, 2.57 ± 0.50) (24 h, S-aphid-infected vs. control treatment, z = 15.87, *p* < 0.05; 24 h, R-aphid-infected vs. control treatment, z = 8.65, *p* < 0.05; 48 h, S-aphid-infected vs. control treatment, z = 8.22, *p* < 0.05; 48 h, R-aphid-infected vs. control treatment, z = 13.94, *p* < 0.05). At the 72nd hour after being infected by two strains of *T. trifolii*, the *AOS* expression level of WL343 had returned to a level which was of no significant difference compared to the blank control.

At the 6th, 24th, and 48th hours after being infected by *T. trifolii*, there was no significant difference in the expression levels of *LOX* (a key synthetic gene of jasmonic acid) of WL343 leaves infected by S-aphids and R-aphids (Figure 3d). At the 6th hour after being infected by two strains of *T. trifolii*, the *LOX* expression level of Wl343 had no significant difference from the blank control. At the 24th and 48th hours after being infected by *T. trifolii*, the *LOX* expression levels of WL343 infected by S-aphids (24 h, 2.44 ± 0.30; 48 h, 2.31 ± 0.26) and R-aphids (24 h, 3.70 ± 0.59; 48 h, 4.42 ± 0.44) were both significantly higher than that of the blank control (24 h, 2.30 ± 0.32; 48 h, 2.15 ± 0.27) (24 h, S-aphid-infected vs. control treatment, z = 7.54, *p* < 0.05; 24 h, R-aphid-infected vs. control treatment, z = 12.94, *p* < 0.05; 48 h, S-aphid-infected vs. control treatment, z = 12.94, *p* < 0.05; 48 h, R-aphid-infected vs. control treatment, z = 16.84, *p* < 0.05). At the 72nd hour after being infected by two strains of *T. trifolii*, the *LOX* expression level of WL343 had returned to a level which had no significant difference compared to the blank control.

At the 6th, 24th, and 48th hours after being infected by *T. trifolii*, there was no significant difference in the expression levels of *ICS* (a key synthetic gene of salicylic acid) of WL343 leaves infected by S-aphids and R-aphids (Figure 3e). At the 6th, 24th and 48th hours after being infected by *T. trifolii*, the *ICS* expression levels of WL343 infected by S-aphids (6 h, 1.72 ± 0.26; 24 h, 2.44 ± 0.30; 48 h, 2.31 ± 0.26) and R-aphids (6 h, 1.75 ± 0.13; 24 h, 3.70 ± 0.59; 48 h, 4.42 ± 0.44) were both significantly higher than that of the blank control (6 h, 1.75 ± 0.13; 24 h, 2.30 ± 0.32; 48 h, 2.15 ± 0.27) (6 h, S-aphid-infected vs. control treatment, z = 5.44, *p* < 0.05; 6 h, R-aphid-infected vs. control treatment, z = 6.1, *p* < 0.05; 24 h, S-aphid-infected vs. control treatment, z = 13.52, *p* < 0.05; 24 h, R-aphid-infected vs. control treatment, z = 11.24, *p* < 0.05; 48 h, S-aphid-infected vs. control treatment, z = 18.52, *p* < 0.05; 48 h, R-aphid-infected vs. control treatment, z = 12.94, *p* < 0.05). At the 72nd hour after being infected by two strains of *T. trifolii*, *ICS* expression level of WL343 had both returned to a level which had no significant difference compared to the blank control.

At the 6th, 24th, and 48th hours after being infected by *T. trifolii*, there was no significant difference in expression level of *PAL* (a key synthetic gene of salicylic acid) of WL343 leaves infected by S-aphids and R-aphids (Figure 3f). At the 6th, 24th, and 48th hours after being infected by *T. trifolii*, the *PAL* expression levels of WL343 infected by S-aphids (6 h, 2.12 ± 0.27; 24 h, 3.28 ± 0.25; 48 h, 2.95 ± 0.34) and R-aphids (6 h, 2.35 ± 0.24; 24 h, 3.44 ± 0.27; 48 h, 3.22 ± 0.50) were both significantly higher than the blank control (6 h, 1.07 ± 0.06; 24 h, 1.05 ± 0.13; 48 h, 1.01 ± 0.09) (6 h, S-aphid-infected vs. control treatment, z = 5.44, *p* < 0.05; 6 h, R-aphid-infected vs. control treatment, z = 8.52, *p* < 0.05; 24 h, S-aphid-infected vs. control treatment, z = 12.56, *p* < 0.05; 24 h, R-aphid-infected vs. control treatment, z = 11.25, *p* < 0.05; 48 h, S-aphid-infected vs. control treatment, z = 13.59, *p* < 0.05; 48 h, R-aphid-infected vs. control treatment, z = 11.56, *p* < 0.05). At the 72nd hour after being infected by two strains of *T. trifolii*, the *PAL* expression level of WL343 had returned to a level which had no significant difference compared to the blank control.

To verify the difference in the ability of R-saliva and S-saliva inducing defense responses in alfalfa. We measured the changes in the content of jasmonic acid and salicylic acid in the leaves of WL343 cultivar alfalfa and Zhongmu No. 1 cultivar alfalfa, as well as the expression levels of key synthetic genes, after being infiltrated by two strains of *T. trifolii*’s saliva (R-saliva and S-saliva).

**Figure 4:**
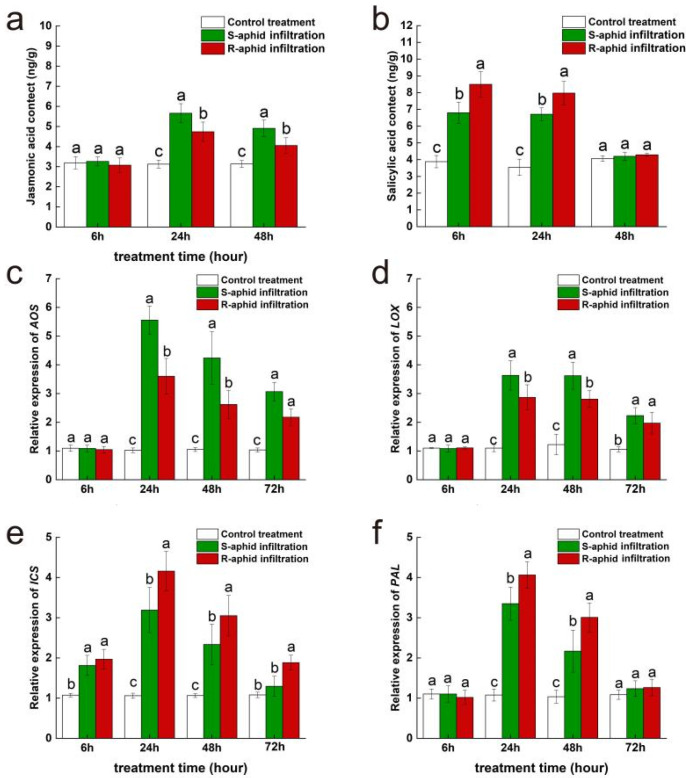
The effect of Zhongmu No. 1 cultivar alfalfa infiltrated by either R-saliva or S-saliva on the subsequent activities of the JA plant hormone (**a**) and SA plant hormone (**b**) signaling pathways and expression of the *AOS* gene (**c**), the *LOX* gene (**d**), the *ICS* gene (**e**), and the *PAL* gene (**f**). The different lowercase letters indicate significant differences in signal hormone contents and gene expression between Zhongmu No. 1 infiltrated by R-saliva or S-saliva (*p* < 0.05; Tukey’s HSD).

At the 24th and 48th hours after being infiltrated by *T. trifolii*’s saliva, the jasmonic acid content of Zhongmu No. 1 was significantly influenced by the strain of *T. trifolii* (24 h: F_2,14_ = 37.71, *p* < 0.05; 48 h: F_2,14_ = 17.02, *p* < 0.05) (Figure 4a). However, at the 6th hour after being infiltrated by two strains of *T. trifolii*’s saliva, the jasmonic acid content of Zhongmu No. 1 had no significant difference from the blank control. Additionally, at the 24th and 48th hours after being infiltrated by *T. trifolii*’s saliva, the jasmonic acid content of alfalfa infiltrated by S-saliva (24 h, 5.66 ± 0.48 mg/g; 48 h, 4.55 ± 0.42 mg/g) was significantly higher than of that infiltrated by R-saliva (24 h, 4.74 ± 0.48 mg/g; 48 h, 3.37 ± 0.38 mg/g) (24 h, z = 3.42, *p* < 0.05; 48 h, z = 2.47, *p* < 0.05), and they both were significantly higher than the blank control (24 h, 3.12 ± 0.13 mg/g; 48 h, 3.14 ± 0.08 mg/g) (24 h, S-saliva infiltration vs. control treatment, z = 5.74, *p* < 0.05; 24 h, R-saliva infiltration vs. control treatment, z = 9.13, *p* < 0.05; 48 h, S-saliva infiltration vs. control treatment, z = 16.75, *p* < 0.05; 48 h, R-saliva infiltration vs. control treatment, z = 10.65, *p* < 0.05).

At the 6th and 24th hours after being infiltrated by *T. trifolii*’s saliva, the salicylic acid content of Zhongmu No. 1 was significantly influenced by the strain of *T. trifolii* (6 h: F_2,14_ = 122.47, *p* < 0.05; 24 h: F_2,14_ = 78.67, *p* < 0.05) (Figure 4b). Additionally, at the 6th and 24th hours after being infiltrated by *T. trifolii*, the salicylic acid content of alfalfa infiltrated by R-saliva (6 h, 8.49 ± 0.76 mg/g; 24 h, 7.97 ± 0.69 mg/g) was significantly higher than that infiltrated by S-saliva (6 h, 6.80 ± 0.76 mg/g; 24 h, 6.71 ± 0.38 mg/g) (6 h, z = 9.24, *p* < 0.05; 24 h, z = 14.72, *p* < 0.05), and they both were significantly higher than the blank control (6 h, 3.88 ± 0.37 mg/g; 24 h, 3.54 ± 0.49 mg/g) (6 h, S-saliva infiltration vs. control treatment, z = 16.52, *p* < 0.05; 6 h, R-saliva infiltration vs. control treatment, z = 14.52, *p* < 0.05; 24 h, S-saliva infiltration vs. control treatment, z = 11.63, *p* < 0.05; 24 h, R-saliva infiltration vs. control treatment, z = 21.41, *p* < 0.05). However, at the 48th hour after being infiltrated by two strains of *T. trifolii*’s saliva, the salicylic acid content of Zhongmu No. 1 had returned to a level which had no significant difference compared to the blank control.

At the 24th and 48th hours after being infiltrated by *T. trifolii*’s saliva, expression levels of *AOS*, a key synthetic gene of jasmonic acid, were significantly influenced by the strain of *T. trifolii* (24 h: F_2,14_ = 102.86, *p* < 0.05; 48 h: F_2,14_ = 42.15, *p* < 0.05) (Figure 4c). At the 6th hour after being infiltrated by two strains of *T. trifolii*’s saliva, the *AOS* expression level of Zhongmu No. 1 had no significant difference from that of the blank control. Additionally, at the 24th and 48th hours after being infiltrated by *T. trifolii*, the *AOS* expression level of Zhongmu No. 1 infiltrated by S-saliva (24 h, 5.56 ± 0.49; 48 h, 4.24 ± 0.91) was significantly higher than of that infiltrated by R-saliva (24 h, 3.60 ± 0.62; 48 h, 2.61 ± 0.49) (24 h, z = 2.94, *p* < 0.05; 48 h, z = 11.52, *p* < 0.05), and they were both significantly higher than the blank control (24 h, 1.05 ± 0.08; 48 h, 1.05 ± 0.07) (24 h, S-saliva infiltration vs. control treatment, z = 21.48, *p* < 0.05; 24 h, R-saliva infiltration vs. control treatment, z = 16.74, *p* < 0.05; 48 h, S-saliva infiltration vs. control treatment, z = 16.74, *p* < 0.05; 48 h, R-saliva infiltration vs. control treatment, z = 11.44, *p* < 0.05). At the 72nd hour after being infected by *T. trifolii*, there was no significant difference in expression level of *AOS* between Zhongmu No. 1 infected by R-aphids (72 h, 2.18 ± 0.28) and S-aphids (72 h, 3.07 ± 0.32), but they were both significantly higher than the blank control (72 h, 1.04 ± 0.07) (72 h, R-saliva infiltration vs. control treatment, z = 20.14, *p* < 0.05; 72 h, S-saliva infiltration vs. control treatment, z = 14.56, *p* < 0.05).

At the 24th and 48th hours after being infiltrated by *T. trifolii*’s saliva, the expression level of *LOX*, a key synthetic gene of jasmonic acid, was significantly influenced by the strain of *T. trifolii* (24 h: F_2,14_ = 102.54, *p* < 0.05; 48 h: F_2,14_ = 52.81, *p* < 0.05) (Figure 4d). At the 6th hour after being infiltrated by two strains of *T. trifolii*’s saliva, the *LOX* expression level of Zhongmu No. 1 had no significant difference from the blank control. Additionally, at the 24th and 48th hours after being infiltrated by *T. trifolii*’s saliva, the *LOX* expression level of Zhongmu No. 1 infiltrated by S-saliva (24 h, 3.63 ± 0.51; 48 h, 3.62 ± 0.47) was significantly higher than of that infiltrated by S-aphids (24 h, 2.86 ± 0.44; 48 h, 3.17 ± 0.29) (24 h, z = 22.56, *p* < 0.05; 48 h, z = 14.63, *p* < 0.05), and they were both significantly higher than the blank control (24 h, 2.86 ± 0.44; 48 h, 3.17 ± 0.29) (24 h, S-saliva infiltration vs. control treatment, z = 13.56, *p* < 0.05; 24 h, R-saliva infiltration vs. control treatment, z = 10.57, *p* < 0.05; 48 h, S-saliva infiltration vs. control treatment, z = 21.43, *p* < 0.05; 48 h, R-saliva infiltration vs. control treatment, z = 15.92, *p* < 0.05). At the 72nd hour after being infiltrated by *T. trifolii*’s saliva, there was no significant difference in expression levels of *LOX* between Zhongmu No. 1 being infiltrated by R-saliva (72 h, 2.23 ± 0.27) and S-saliva (72 h, 1.97 ± 0.38), but they were both significantly higher than the blank control (72 h, 1.05 ± 0.09) (72 h, R-saliva infiltration vs. control treatment, z = 22.65, *p* < 0.05; 72 h, S-saliva infiltration vs. control treatment, z = 13.65, *p* < 0.05).

At the 24th, 48th, and 72nd hours after being infiltrated by *T. trifolii*’s saliva, expression levels of *ICS*, a key synthetic gene of salicylic acid was significantly influenced by the strain of *T. trifolii* (24 h: F2,14 = 66.74, *p* < 0.05; 48 h: F2,14 = 34.14, *p* < 0.05; 48 h: F2,14 = 22.62, *p* < 0.05) (Figure 4e). At the 6th hour after being infiltrated by *T. trifolii*’s saliva, there was no significant difference in the expression levels of *ICS* between Zhongmu No. 1 being infiltrated by R-saliva (6 h, 1.97 ± 0.24) and S-saliva (6 h, 1.81 ± 0.25), but they were both significantly higher than the blank control (6 h, 1.07 ± 0.04) (6 h, R-saliva infiltration vs. control treatment, z = 13.45, *p* < 0.05; 6 h, S-saliva infiltration vs. control treatment, z = 9.14, *p* < 0.05). At the 24th, 48th, and 48th hour after being infiltrated by *T. trifolii*’s saliva, *ICS* expression level of Zhongmu No. 1 infiltrated by R-saliva (24 h, 4.16 ± 0.49; 48 h, 3.05 ± 0.51; 72 h, 1.88 ± 0.18) was significantly higher than that infiltrated by S-saliva (24 h, 3.39 ± 0.56; 48 h, 2.74 ± 0.51; 72 h, 1.59 ± 0.25) (24 h, z = 16.52, *p* < 0.05; 48 h, z = 16.94, *p* < 0.05; 72 h, z = 11.95, *p* < 0.05). At the 6th, 24th, and 48th hour after being infiltrated by two strains *T. trifolii*’s saliva, *ICS* expression level of Zhongmu No. 1 were significantly higher than the blank control (6 h, 1.07 ± 0.04; 24 h, 1.06 ± 0.06; 48 h, 1.06 ± 0.05) (6 h, S-saliva infiltration vs. control treatment, z = 12.11, *p* < 0.05; 6 h, R-saliva infiltration vs. control treatment, z = 13.56, *p* < 0.05; 24 h, S-saliva infiltration vs. control treatment, z = 18.54, *p* < 0.05; 24 h, R-saliva infiltration vs. control treatment, z = 11.94, *p* < 0.05; 48 h, S-saliva infiltration vs. control treatment, z = 11.25, *p* < 0.05; 48 h, R-saliva infiltration vs. control treatment, z = 13.94, *p* < 0.05). However, at the 72nd hour after being infiltrated by *T. trifolii*’s saliva, only the *ICS* expression level of Zhongmu No. 1 infiltrated by R-saliva (72 h, 1.84 ± 0.2) was significantly higher than the blank control (72 h, 1.08 ± 0.07) (72 h, R-saliva infiltration vs. control treatment, z = 22.64, *p* < 0.05); the *ICS* expression level of Zhongmu No. 1 infiltrated by S-saliva had returned to a level which had no significant difference compared to the blank control.

At the 24th and 48th hour after being infiltrated by *T. trifolii*’s saliva, expression level of *PAL*, a key synthetic gene of salicylic acid was significantly influenced by the strain of *T. trifolii* (24 h: F_2,14_ = 181.23, *p* < 0.05; 48 h: F_2,14_ = 16.05, *p* < 0.05) (Figure 4f). At the 6th hour after being infiltrated by *T. trifolii*’s saliva, there was no significant difference in expression level of *PAL* between Zhongmu No. 1 being infiltrated by R-saliva and S-saliva, and neither of them were significantly different from the blank control. At the 24th and 48th hour after being infiltrated by *T. trifolii*, *PAL* expression level of Zhongmu No. 1 infiltrated by R-saliva (24 h, 4.06 ± 0.33; 48 h, 2.41 ± 0.36) was significantly higher than infiltrated by S-saliva (24 h, 3.35 ± 0.41; 48 h, 2.17 ± 0.52) (24 h, z = 16.84, *p* < 0.05; 48 h, z = 11.23, *p* < 0.05), and they were both significantly higher than the blank control (24 h, 1.18 ± 0.12; 48 h, 1.06 ± 0.13) (24 h, S-saliva infiltration vs. control treatment, z = 12.05, *p* < 0.05; 24 h, R-saliva infiltration vs. control treatment, z = 8.61, *p* < 0.05; 48 h, S-aphid-infected vs. control treatment, z = 11.27, *p* < 0.05; 48 h, R-saliva infiltration vs. control treatment, z = 12.94, *p* < 0.05). However, at the 72nd hour after being infiltrated by two strains of *T. trifolii*’s saliva, *PAL* expression level of Zhongmu No. 1 returned to a level which had no significant difference compared to the blank control.

**Figure 5:**
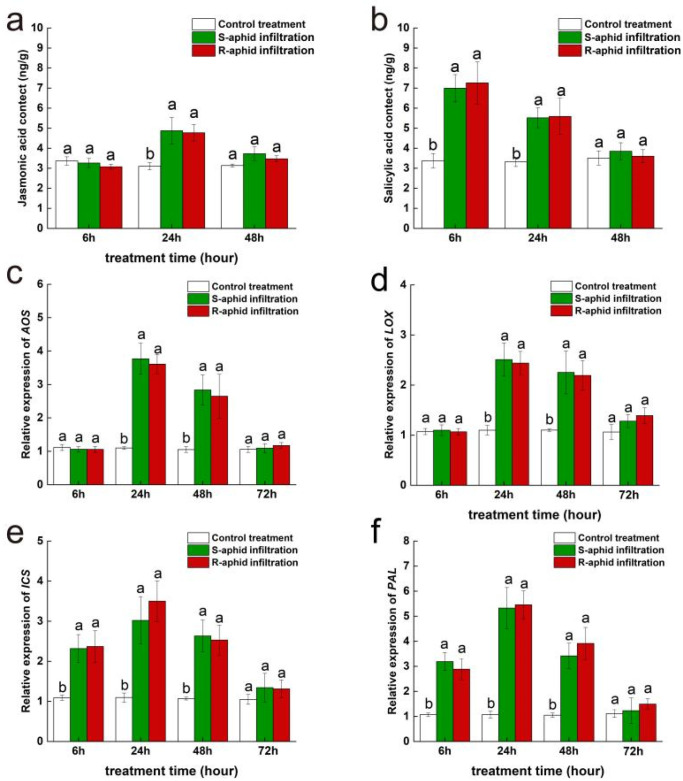
The effect of WL343 cultivar alfalfa infiltrated by either R-saliva or S-saliva on the subsequent activities of the JA plant hormone (**a**) and SA plant hormone (**b**) signaling pathways, and expression of the *AOS* gene (**c**), the *LOX* gene (**d**), the *ICS* gene (**e**), and the *PAL* gene (**f**). The different lowercase letters indicate significant differences in signal hormone contents and gene expression between Zhongmu No. 1 infiltrated by R-saliva or S-saliva (*p* < 0.05; Tukey’s HSD).

At the 6th, 24th, and 48th hours after being infiltrated by *T. trifolii*’s saliva, there was no significant difference in jasmonic acid content between WL343 leaves infiltrated by S-saliva and R-saliva (Figure 5a). At the 6th hour after being infiltrated by two strains of *T. trifolii*’s saliva, the jasmonic acid content of Wl343 was not significantly different from the blank control. At the 24th hour after being infiltrated by *T. trifolii*, the jasmonic acid contents of WL343 infiltrated by S-aphids (24 h, 4.87 ± 0.66) and R-aphids (24 h, 4.77 ± 0.41) were both significantly higher than the blank control (24 h, 3.10 ± 0.18) (24 h, S-saliva infiltration vs. control treatment, z = 15.94, *p* < 0.05; 24 h, R-saliva infiltration vs. control treatment, z = 15.64, *p* < 0.05).

At the 6th, 24th, and 48th hour after being infiltrated by *T. trifolii*’s saliva, there was no significant difference in salicylic acid content between WL343 leaves infiltrated by S-saliva and R-saliva (Figure 5b). At the 6th and 24th hours after being infiltrated by *T. trifolii*’s saliva, the salicylic acid content of WL343 infiltrated by S-saliva (6 h, 7.00 ± 0.68 mg/g; 24 h, 5.52 ± 0.50 mg/g) and R-saliva (6 h, 7.26 ± 1.06 mg/g; 24 h, 5.59 ± 0.90 mg/g) were both significantly higher than the blank control (6 h, 3.37 ± 0.36 mg/g; 24 h, 3.32 ± 0.23 mg/g) (6 h, S-saliva infiltration vs. control treatment, z = 13.54, *p* < 0.05; 6 h, R-saliva infiltration vs. control treatment, z = 11.85, *p* < 0.05; 24 h, S-saliva infiltration vs. control treatment, z = 9.14, *p* < 0.05; 24 h, R-saliva infiltration vs. control treatment, z = 12.65, *p* < 0.05). However, at the 48th hour after being infiltrated by two strains of *T. trifolii*’s saliva, the salicylic acid content of WL343 had returned to a level which had no significant difference compared to the blank control.

At the 6th, 24th, and 48th hours after being infiltrated by *T. trifolii*’s saliva, there was no significant difference in the expression level of *AOS* (a key synthetic gene of jasmonic acid) of WL343 leaves infiltrated by S-saliva and R-saliva (Figure 5c). At the 6th hour after being infiltrated by two strains of *T. trifolii*’s saliva, the *AOS* expression levels of Wl343 have no significant difference from the blank control. At the 24th and 48th hours after being infiltrated by *T. trifolii*’s saliva, the *AOS* expression levels of WL343 infiltrated by S-saliva (24 h, 3.77 ± 0.47; 48 h, 2.84 ± 0.45) and R-saliva (24 h, 3.61 ± 0.29; 48 h, 2.65 ± 0.64) were both significantly higher than the blank control (24 h, 1.10 ± 0.04; 48 h, 1.05 ± 0.09) (24 h, S-saliva infiltration vs. control treatment, z = 10.65, *p* < 0.05; 24 h, R-saliva infiltration vs. control treatment, z = 16.84, *p* < 0.05; 48 h, S-saliva infiltration vs. control treatment, z = 13.64, *p* < 0.05; 48 h, R-saliva infiltration vs. control treatment, z = 11.08, *p* < 0.05). At the 72nd hour after being infiltrated by *T. trifolii*’s saliva, the *AOS* expression level of WL343 had returned to a level which had no significant difference compared to the blank control.

At the 6th, 24th, 48th, and 72nd hours after being infiltrated by *T. trifolii*’s saliva, there was no significant difference in the expression levels of *LOX* (a key synthetic gene of jasmonic acid) of WL343 leaves infiltrated by S-saliva and R-saliva (Figure 5d). At the 6th hour after being infiltrated by two strains of *T. trifolii*’s saliva, the *LOX* expression level of Wl343 had no significantl difference from the blank control. At the 24th and 48th hours after being infiltrated by *T. trifolii*’s saliva, the *LOX* expression levels of WL343 infiltrated by S-saliva (24 h, 2.51 ± 0.33; 48 h, 2.25 ± 0.43) and R-aphids (24 h, 2.44 ± 0.23; 48 h, 2.19 ± 0.30) were both significantly higher than the blank control (24 h, 1.10 ± 0.10; 48 h, 1.10 ± 0.02) (24 h, S-saliva infiltration vs. control treatment, z = 5.64, *p* < 0.05; 24 h, R-saliva infiltration vs. control treatment, z = 12.94, *p* < 0.05; 48 h, S-saliva infiltration vs. control treatment, z = 3.94, *p* < 0.05; 48 h, R-saliva infiltration vs. control treatment, z = 15.26, *p* < 0.05). At the 72nd hour after being infiltrated by two strains of *T. trifolii*’s saliva, the *LOX* expression level of WL343 returned to a level which had no significant difference compared to the blank control.

At the 6th, 24th, 48th, and 72nd hours after being infiltrated by *T. trifolii*’s saliva, there was no significant difference in the expression levels of *ICS* (a key synthetic gene of salicylic acid) of WL343 leaves infiltrated by S-saliva and R-saliva (Figure 5e). At the 6th, 24th, and 48th hours after being infiltrated by *T. trifolii*’s saliva, *ICS* expression level of WL343 infiltrated by S-aphids (6 h, 2.32 ± 0.35; 24 h, 3.02 ± 0.59; 48 h, 2.63 ± 0.40) and R-saliva (6 h, 2.37 ± 0.39; 24 h, 3.50 ± 0.51; 48 h, 2.52 ± 0.37) were both significantly higher than the blank control (6 h, 1.09 ± 0.06; 24 h, 1.10 ± 0.11; 48 h, 1.07 ± 0.03) (6 h, S-saliva infiltration vs. control treatment, z = 15.84, *p* < 0.05; 6 h, R-saliva infiltration vs. control treatment, z = 20.44, *p* < 0.05; 24 h, S-saliva infiltration vs. control treatment, z = 11.94, *p* < 0.05; 24 h, R-saliva infiltration vs. control treatment, z = 13.29, *p* < 0.05; 48 h, S-saliva infiltration vs. control treatment, z = 23.15, *p* < 0.05; 48 h, R-saliva infiltration vs. control treatment, z = 10.95, *p* < 0.05). At the 72nd hour after being infiltrated by two strains of *T. trifolii*’s saliva, the *ICS* expression level of WL343 returned to a level which had no significant difference compared to the blank control.

At the 6th, 24th, 48th, and 72nd hours after being infiltrated by *T. trifolii*’s saliva, there was no significant difference in the expression levels of *PAL* (a key synthetic gene of salicylic acid) of WL343 leaves infiltrated by S-saliva and R-saliva (Figure 5f). At the 6th, 24th, and 48th hours after being infiltrated by *T. trifolii*’s saliva, the *PAL* expression level of WL343 infiltrated by S-saliva (6 h, 3.19 ± 0.36; 24 h, 5.32 ± 0.82; 48 h, 3.42 ± 0.50) and R-saliva (6 h, 2.89 ± 0.42; 24 h, 5.46 ± 0.56; 48 h, 3.91 ± 0.64) were both significantly higher than the blank control (6 h, 1.07 ± 0.06; 24 h, 1.07 ± 0.13; 48 h, 1.05 ± 0.09) (6 h, S-saliva infiltration vs. control treatment, z = 13.26, *p* < 0.05; 6 h, R-saliva infiltration vs. control treatment, z = 26.74, *p* < 0.05; 24 h, S-saliva infiltration vs. control treatment, z = 14.65, *p* < 0.05; 24 h, R-saliva infiltration vs. control treatment, z = 21.95, *p* < 0.05; 48 h, S-saliva infiltration vs. control treatment, z = 21.95, *p* < 0.05; 48 h, S-saliva infiltration vs. control treatment, z = 12.94, *p* < 0.05). At the 72nd hour after being infiltrated by two strains of *T. trifolii*’s saliva, the *PAL* expression level of WL343 returned to a level which had no significant difference compared to the blank control.

## 3. Discussion

Over the course of millions of years of coevolution, plants have developed a diverse array of defense mechanisms aimed at mitigating or preventing damage caused by insects [20]. In reaction to the defensive mechanisms employed by plants, herbivorous insects have likewise adapted and are continually evolving strategies that enable them to exploit plant resources effectively [21]. In reaction to the defensive mechanisms employed by plants, herbivorous insects have likewise adapted and are continually evolving strategies that enable them to exploit plant resources effectively [22].

The capacity of *T. trifolii* to withstand the defensive mechanisms of alfalfa exhibited variability depending on the specific cultivar of alfalfa on which it was reared. The findings of this study indicated that there were no statistically significant differences in the survival rates or population growth of the R-aphid and S-aphid populations when they were cultivated on the aphid-susceptible alfalfa cultivar (WL343). However, In the case of aphid-resistant alfalfa (Zhongmu No. 1), the survival and growth rates of the R-aphids were markedly superior to those of S-aphids (Figure 1). Numerous instances have been documented in which monophagous insects have adapted to various cultivars of host plants, resulting in significant phenotypic variation among these insects. For instance, the concentrations of trehalose, choline metabolites, and nucleotides were markedly elevated in *Nilaparvata lugens* that were feeding on the insect-susceptible rice cultivar Taichung native (TN1) compared to those feeding on the insect-resistant cultivar TN1, which possesses the *BPH15* insect-resistant gene [23]. Similarly, Chen demonstrated that the transcriptomic profiles of *Mayetiola destructor* larvae cultivated on insect-resistant wheat (cv. Molly) differed significantly from those reared on insect-susceptible wheat (cv. Newton) [24]. Consequently, we hypothesized that the concentrations of detoxifying enzymes, proteolytic enzymes, and various genes associated with plant defense mechanisms would exhibit a significant increase in R-aphids after multiple generations of selection on aphid-resistant alfalfa. In contrast, the survival rate of aphids that were not subjected to the selection process was found to be lower when they were fed on aphid-resistant alfalfa.

The variation in salivary composition is the underlying factor that accounts for the differing levels of adaptability exhibited by R-aphids and S-aphids towards their respective host plants. In contrast to S-aphids, R-aphids possess the capability to diminish the resistance of host plants to aphid infestation and enhance their own survival rates on these plants through the utilization of salivary proteins. The findings of our study indicated that in comparison to plants infiltrated with S-saliva and the control group, aphids exhibited a greater preference for feeding on host plants infiltrated with R-saliva. Through the assessment of survival rates of native aphids on host plants treated with various salivary extracts, our findings indicate that the mortality risk for aphids on host plants infiltrated with R-saliva was markedly lower compared to those on host plants treated with S-saliva and the blank control. The mechanisms underlying plant resistance to insect herbivory can be primarily classified into two categories. The first category is characterized by the jasmonic acid (JA) signaling pathway, which facilitates the synthesis of secondary metabolites, including nitrogenous compounds and phenolic compounds. These metabolites serve to deter insects by exerting toxic effects, thereby conferring direct resistance to the plants [25].

In the present study, we observed that aphids initially commence feeding on any given leaf; however, a subset of aphids subsequently avoids that leaf and transitions to feeding on other leaves subjected to different treatments. After a duration of 24 h, the majority of aphids exhibited a reluctance to alter their host. We hypothesized that the host selection behavior of aphids in this experiment was predominantly influenced by the gustatory properties of the host during feeding rather than olfactory cues utilized in the host’s location. Furthermore, the R-saliva infiltration treatment may disrupt the synthesis of secondary metabolites that are downstream of the jasmonic acid (JA) signaling pathway in the host plants. Urbanska discovered that the enzyme 1,4-glucosidase, present in aphid saliva, has the potential to facilitate the degradation of phenolic compounds, which are known to be toxic to aphids [26]. Numerous studies have documented strategies aimed at enhancing insect survival rates through the suppression of the defensive mechanisms of host plants. For instance, Michaud’s research indicates that the presence of *Ceratovacuna lanigera* can facilitate the survival, growth, and reproductive success of *Rhopalosiphum padi* when these aphids infest sorghum [27]. More research is required to determine the molecular mechanism by which R-saliva prevents the production of secondary metabolites in plants. As a result, we also looked at the amounts of SA plant hormone and JA plant hormone plant hormone production in host plants following aphid infection or salivary penetration.

**Figure 6:**
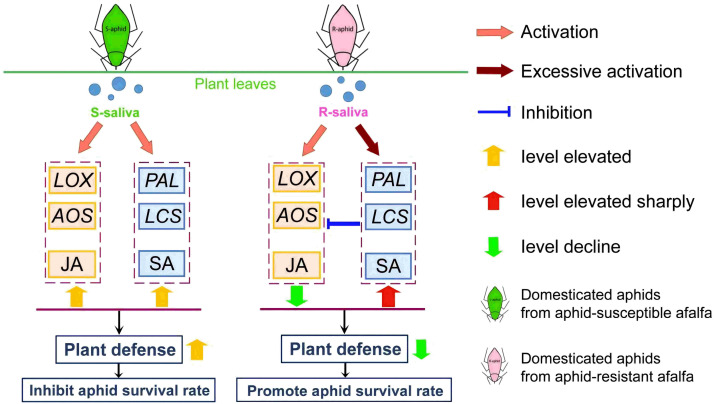
Schematic diagram of changes in gene expression levels related to salicylic acid (SA) plant hormone and jasmonic acid plant hormone (JA) synthesis in host plant leaves after infestation by *T. trifolii*. When S-aphids infest the leaves of the host plant, the SA plant hormone and JA plant hormone signal pathways of the host plant are activated at the same time; they improve the host plant’s ability to resist the aphids together and then reduce the survival rate of the aphids. However, when R-aphids infested the leaves of the host plant, the SA plant hormone signal pathway is activated excessively, resulting in a sharp increase in SA plant hormone synthesis. The JA plant hormone pathway in the host plant is consequently inhibited, the host plant’s ability to resist aphids is reduced, and the survival rate of the aphids increases.

Salivary protein could activate SA plant hormone synthesis and inhibit JA plant hormone synthesis as a means for the R-aphids to interfere with plant defenses. The results showed that the JA plant hormone synthesis level in host plants infested by R-aphids was lower than in plants infested by S-aphids. In comparison, the level of SA plant hormone synthesis was significantly higher than in plants fed on by S-aphids. The same results were obtained after the host plants were infiltrated with the saliva of these two aphids. These results indicate that the salivary proteins secreted by the aphids when they infested the host plants were the effectors of the aphids in regulating the plant defense response. This finding was similar to the study by Cui, in which the *Armet* (arginine-rich) protein gene in the salivary proteins of *Acyrthosiphon pisum* (Harris) was transiently expressed in *Nicotiana benthamiana* and *M. truncatula*, and the host plant SA plant hormone significantly increased while the JA plant hormone signal pathway was weakened [28]. Xu reported that *Bemisia tabaci* could secrete a small molecular weight protein, BT56, that overstimulated the SA plant hormone pathway of the host plant (*N. benthamiana*) and promoted its feeding [29]. Tian identified glucose oxidase (*GOX*) in the saliva of *Spodoptera frugiperda* (Smith) larvae, which could also inhibit the production of host plant JA plant hormone [30]. Therefore, the R-saliva in this study could inhibit the JA plant hormone pathway by activating the plant SA plant hormone pathway, thereby reducing the host plant’s defensive ability and improving the insect’s fitness (Figure 6). However, there are still unexplainable biological phenomena in this study. For instance, research by He indicated that the function of plant phenylalanine ammonia-lyase (PAL) genes extends beyond the biosynthesis of salicylic acid (SA); it also plays a significant role in the synthesis of lignin and flavonoids, which exhibit direct toxic effects on insect pests, including aphids [31]. This finding stands in contrast to the conclusions presented in this article. The detoxification mechanisms employed by *T. trifolii* to mitigate the effects of toxic compounds produced by alfalfa require additional investigation. In addition, the effectors in the aphid salivary proteins need to be further verified. The results of this study provide crucial support for revealing the mechanisms by which host plants regulate pest defense.

## 4. Materials and Methods

### 4.1. Plant Material

According to the results of initial laboratory field trials, in the conducted research, our laboratory investigated the variations in aphid population density across 28 different alfalfa cultivars through field experiments aimed at identifying insect-resistant alfalfa cultivars. The results indicated that the aphid population density on the Zhongmu No. 1 cultivar was significantly lower, recorded at 773 individuals per 100 branches, whereas the WL343 variety exhibited a markedly higher population density, with 3126 individuals per 100 branches [32]. Therefore, two cultivars of *M. sativa* were selected for further study: cv. Zhongmu No. 1, characterized by its resistance to aphids, and cv. WL343, which exhibits susceptibility to aphids. Prior to planting, seeds from both cultivars were subjected to a soaking process in warm water at a temperature of 25 °C for a duration of 6 h. Seeds were sown at a depth of 2 cm within rectangular flowerpots measuring 40 cm in length, 30 cm in width, and 7 cm in depth, which were filled with a commercially available potting mix. Each cultivar was represented by five replicates, with each pot containing approximately 100 uniform seeds. The potting mix was formulated using a combination of peat and coconut bran, sourced from Mandelai Co., located in Qingdao, Shandong Province, China. The experimental pots were arranged within a controlled climate chamber (Xunneng Ltd. Co., Jinan, China, Model PGC-450) maintained at a temperature range from 25 to 27 °C, relative humidity of 80 to 85%, and a light/dark cycle of 16 h of light and 8 h of darkness. Aphids were introduced to the plants when they reached 16 days of age. At this juncture, alfalfa has attained the initial three-leaf stage, exhibiting an average plant height of 15 ± 2 cm.

### 4.2. Aphid Cultures

The *T. trifolii* population, initially sourced from *M. sativa* in Cangzhou, Hebei province, was cultivated at the Beijing Institute of Plant Protection, Chinese Academy of Agricultural Sciences. *T. trifolii* utilized in the experiments were cultivated on alfalfa (Wl343) under controlled indoor laboratory conditions for over 100 generations. Prior to their domestication, all *T. trifolii* were provided with an artificial diet for a duration of 5 days. The liquid diet was developed based on the formulation established by Auclair and Cartier, which comprised vitamins, amino acids, sucrose, and various trace elements essential for the survival of aphids [33]. The diet was made available to the aphids via a double-layered Parafilm M membrane [34]. Aphids were allocated to 100 rearing chambers, with each chamber having the capacity to accommodate approximately 200 aphids for the purpose of feeding. The primary element of the rearing chamber consisted of a transparent glass tube measuring 3 cm in diameter and 5 cm in height. To prevent the aphids from escaping, one end of the tube was sealed with sterile cotton, which permitted adequate air circulation. A volume of 100 µL of the diet was positioned within a layer of transparent sealing film, which was subsequently wrapped over the opening to secure the opposite end. Subsequently, the liquid was confined between two layers through the application of an additional layer of stretched sealing film. In the bioassay designed to assess aphid growth and survival, 500 or more aphids were transferred from artificial feed to two different alfalfa cultivars, Zhongmu 1 and WL343. Following 50 generations of exposure to various alfalfa cultivars, *T. trifolii* cultivated on Zhongmu No. 1 were classified as resistant lines (R-aphids), whereas those reared on WL343 were characterized as susceptible lines (S-aphids).

### 4.3. Aphid Survival on the Two Alfalfa Cultivars

Twelve transparent plexiglass tubes measuring 30 cm in height and 3 cm in diameter were positioned within rectangular containers with dimensions of 40 cm in length, 30 cm in width, and 7 cm in depth, which were filled with potting soil. Each tube contained a single seed, which was enveloped in potting mix to a depth of 3 to 4 cm. A total of 240 pots were cultivated for each cultivar. Nine time intervals (0, 2, 6, 12, 24, 48, 72, 96, and 120 h) were randomly allocated to the tubes, with five replicates conducted for each interval. A time interval of 0 h served as the control. The pots were then relocated to a climate-controlled chamber (Model PGC-450, Xunneng Ltd. Co.), where the temperature was maintained at 25 °C with a light:dark cycle of 14:10 to facilitate further experiments and assessments regarding the penetration of aphid saliva.

Following a period exceeding 50 generations, around 2000 mature aphids, which were collected within 12 h post-molt, were selected from each of the two cultivars. The R-aphids and S-aphids were inoculated onto the Zhongmu No. 1 and WL343 cultivars in a uniform manner, with aphid-free plants serving as control specimens. At the time of inoculation, the plants were 15 days old, and each replicate for the aphid treatments received an addition of 30 aphids.

The experimental treatments consisted of six conditions; refer to Table 1 for comprehensive information.

In order to prevent the escape of aphids following inoculation, sterile cotton was employed to seal the tubes. Bioassays commenced 1 h following the introduction of the aphids. Subsequent sampling of aphids was conducted at intervals of 0, 2, 6, 12, 24, 48, 72, 96, and 120 h. At specified intervals, the plants were removed from the tubes and all aphids were carefully transferred into a glass Petri dish (8 cm in diameter) utilizing a fine brush. Following this, the aphids were counted after a thorough examination of the tube walls. The survival rate of the aphids was determined by the formula:

(Number of surviving adult aphids/30) × 100%.

Whole plants that had been subjected to aphid infestation for durations of 6, 24, 48, and 72 h were collected to evaluate the effects of aphid exposure on hormone levels and associated gene expression in the two cultivars. At each designated time point, 5 replicates from each treatment group were randomly chosen, culminating in a total of 20 plants for each time interval. After the roots were excised, any remaining potting mix adhering to the leaves was removed through rinsing with distilled water. Plants were submerged in liquid nitrogen and manually ground into a powder with a pestle and mortar made of ceramic materials. Two portions of 0.100 g of powder for each sample were weighed, placed into separate 2 mL centrifuge tubes, and kept at −80 °C until analysis. In order to measure hormone content and gene expression, a total of 120 plants (20 plants per treatment) were collected.

### 4.4. Collection of T. trifolii Saliva

Saliva samples were obtained by permitting aphids, derived from either the R-aphid or S-aphid cultures, to feed on a 10% sucrose solution. Following the removal of the aphids, the resultant fluid was collected. A sterile glass tube measuring 4 cm in length and 2.5 cm in diameter was sealed at one end with a layer of Parafilm M, onto which 100 µL of sterile artificial diet was applied. In the process of fabricating a double-layer film, an additional layer of Parafilm M was applied over the sucrose solution. Subsequently, each tube was populated with 100 mature aphids that had undergone a 24-h starvation period, and sterile cotton was utilized to seal the tubes. Concurrently, an identical experimental configuration was established, wherein 100 µL of sterile artificial diet was introduced into the double-layer Parafilm M film. However, no aphids were included in the glass tube, thereby designating this setup as a blank control treatment for subsequent experiments. Following the feeding of the aphids, both the tubes containing the aphids and those without were placed in an environmental chamber for a duration of 24 h (at T = 25–27 °C, RH = 80–85%, and L:D = 16 h:8 h).

Aphids were attracted to the sucrose solution contained within the tube, which was partially obscured by black cloth at one end, while the opposite end was oriented towards an incandescent light source within the environmental chamber. A total of 1000 tubes were allocated for each treatment group—R-aphid, S-aphid, and a control group without aphids. Each test tube was populated with approximately 100 aphids. After a duration of 24 h, the tubes were extracted, and the solution located within the central region of the double-layer membrane was collected using a 200 mL range syringe. Subsequently, the sucrose solutions corresponding to each treatment were combined and transferred into a 200 mL conical container. Each conical container was filled with approximately 100 mL of sucrose solutions, both with and without the presence of aphids for feeding purposes. Subsequently, an equal volume of a 2% SDS Tris solution (pH = 8) was incorporated into the sucrose solution, thereby stabilizing the pH of the mixture at approximately neutral levels due to the properties of the SDS Tris solution. The protein concentration within the solution will be determined in a subsequent step.

The combined solution from each treatment was subsequently placed into an ultrafiltration tube with a molecular weight cut-off (MWCO) of 3000. Following this, the mixture underwent centrifugation for a duration of 120 min at a speed of 5000 rpm (2500 g) and a temperature of 4 °C, utilizing a centrifuge (Model 3K15, Sigma Ltd. Co. Martin Christ Gefriertrocknungsanlagen GmbH, Berlin, Germany). The three types of solutions were concentrated to a volume of 5 mL and subsequently aliquoted into 1.5 mL centrifuge tubes prior to conducting the bioassays. The saliva sample obtained from the R-aphids was labeled as “R-saliva”, while the saliva sample from the S-aphids was referred to as “S-saliva”. Additionally, the solution that did not involve aphid feeding was designated as “the blank control”.

### 4.5. Treatment of Alfalfa Leaves with Aphid Saliva

For this study, 30-day-old Zhongmu No. 1 and WL343 cultivars of alfalfa served as the host plants, with a total of 100 plants designated for each treatment group. The upper three leaves of each alfalfa plant were selected for the infiltration treatment. The saliva solution from each treatment was diluted to 10 times its original volume using distilled water, after which 100 µL of the diluted saliva solution was introduced into the interior of the leaves using a 100 µL syringe.

Following the infiltration of the saliva solution into the leaves, the plants—designated as R-saliva infiltrated, S-saliva infiltrated, and the blank control—were cultivated in a controlled environment chamber maintained at a temperature of 25 °C to 27 °C, relative humidity of 80% to 85%, and a light/dark cycle of 16 h light and 8 h dark. This growth period continued until the plants were utilized for the assessment of defense gene expression and the quantification of defense substance content.

In order to investigate the impact of saliva on hormone levels and associated gene expression, a total of five plants were randomly selected from each of the three groups: R-saliva infiltration, S-aphid infiltration, and blank infiltration (the composite solution comprising sodium dodecyl sulfate (SDS), Tris buffer, sucrose, water, and additional components). The selections were conducted at four specific time intervals: 6, 24, 48, and 72 h. Consequently, a total of 15 plants were examined for each time point, resulting in an overall sample size of 60 plants for the study.

A scalpel was utilized to excise the roots, while distilled water was employed to eliminate any remaining potting mix from the leaves. The plants were then immersed in liquid nitrogen and ground into a fine powder. To create two technical replicates for each plant, 0.2 g of the powdered material was precisely measured using an analytical balance and evenly distributed into two 2 mL centrifuge tubes. The resulting powder was stored at −80 °C for subsequent analysis.

### 4.6. Aphid Survival Rate on Saliva-Infiltrated Leaves

Aphids were selected for study within a timeframe of less than 12 h following their transition to the adult stage. In total, 30 aphids were removed from the leaves of 45 plants that had been infiltrated by either R-saliva, S-saliva, or the blank control using a small brush. Aphids were positioned on three leaves of each plant, with roughly 10–12 aphids per leaf. Then, three treatments were formed: aphids on alfalfa infiltrated with R-saliva, aphids on alfalfa infiltrated with S-saliva, and aphids on alfalfa infiltrated with the blank control.

The host plants infested by the aphids for 0, 2, 6, 12, 24, 48, 72, 96, and 120 h were collected separately after the aphids had been allowed to settle on the plants for 1 h. For every time point, three plants of each treatment were collected. The aphids from each plant were moved to a plastic Petri dish (D = 8 cm) with a small brush. The walls of the tubes and the surface of the potting mix were examined closely to ensure that there were no aphids missing. Aphid survival rate was calculated as the number of surviving adult aphids/30 × 100%. Every morning at 9:00, assessments were made for the treatments of the 12 to 96 h time intervals.

### 4.7. Aphid Preferences for the Host Plants After Saliva Infiltration Treatment

**Figure 7:**
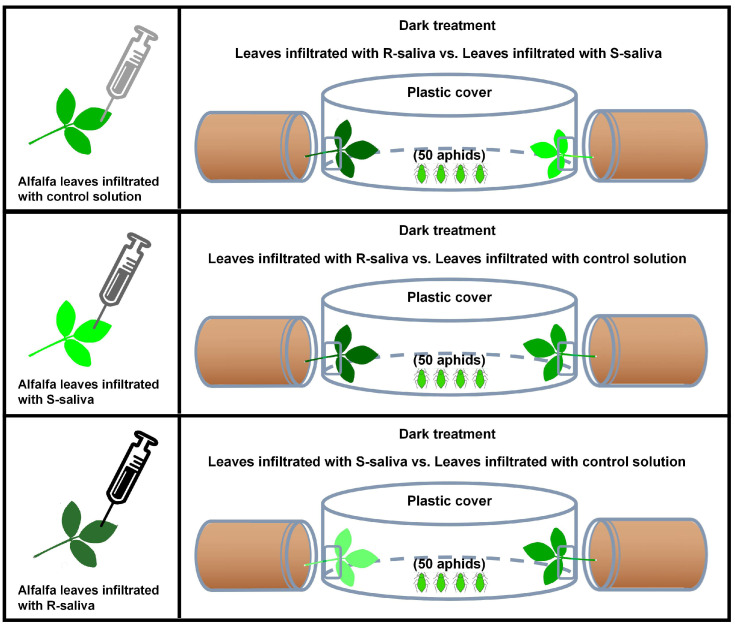
Demonstration of aphid dual-choice experiment device.

As the experimental materials for this section, the adult aphids (less than 12 h after molting to adults) were originally feeding on the alfalfa of WL343, and the aphids were fed with artificial diet after 3rd instar (Figure 7). The feeding time for the artificial diet was approximately 7 days. As test platforms, disposable plastic culture dishes (1.5 cm × 10 cm (H × D)) had two square holes punched into them at each end using sterile scissors (1 cm × 1 cm (L × W)). The host plants were laid out flat on the experimental dishes following the infiltration treatment. The length of the plants that extended into both holes was 3 cm, and the new leaves were inserted into the Petri dishes through the opening.

This experimental design allowed for three comparisons: host plants infiltrated with R-saliva versus host plants infiltrated with S-saliva; host plants infiltrated with R-saliva versus host plants infiltrated with control solution, and host plants infiltrated with S-saliva versus host plants infiltrated with control solution. Using a very small brush, 50 mature adult aphids that had been collected in a 1.5 mL centrifuge tube were placed into the center of the Petri dish. The numbers of aphids on the two sides of the leaves were counted after 6, 24, and 48 h. Each treatment was replicated 10 times, and the positions of the treatment groups on both sides were exchanged at each assessment time point. Aphid preference rate (%) = The number of aphids residing on a treated leaf/50 × 100%.

### 4.8. Determination of Salicylic Acid Plant Hormones and Jasmonic Acid Plant Hormones Content

Plants (on the basis of uniformity regarding growth stage) were selected at different time periods starting at time 0 (aphids infected or after saliva infiltrated) and then at 2, 6, 12, 24, 48, 72, 96, and 120 h after the addition of aphids or saliva infiltration. Six plants (six replicates) were collected at each time period from each treatment. The aphids were carefully collected from each plant with a soft-bristle artist’s brush, and the plant roots were separated from the stem with a scalpel. An aliquot of each replicate (each replicate included most of leaves and small part of stems) was placed into a 1.5 mL centrifuge tube. Finally, the foliage was placed in liquid nitrogen, meshed into a powder, and stored at −80 °C prior to further processing.

ELISA kits were used to determine the salicylic acid (SA) plant hormone and jasmonic acid (JA) plant hormone levels in the two cultivars of alfalfa. Each test used 0.1 g of plant materials (plant powder).

The determination method of salicylic acid plant hormones refers to the instructions of Elisa reagent kit (Cat. SP29771). HRP (horse radish peroxide) was used as the reaction substrate and incubated at 37 °C for 30 min. The OD value was measured at a wavelength of 450 nm using an enzyme-linked immunosorbent assay (Multiskan FC). Finally, the concentration of salicylic acid plant hormones in alfalfa leaves was calculated using the previously determined linear regression equation.

The determination method of jasmonic acid plant hormone refers to the instructions of the Elisa reagent kit (Cat. SP2938). The measurement method is similar to the salicylic acid plant hormone content method except that the substrate is changed to TMB (3,3′, 5,5′—Tetramethylbenzidine). Finally, the concentration of jasmonic acid plant hormone in alfalfa leaves is calculated using the previously determined linear regression equation.

### 4.9. Extraction of Total RNA, Synthesis of cDNA, and RT-qPCR Analysis

The ground alfalfa leaves that had been exposed to R-aphid infestation, S-aphid infestation, no aphid infestation, R-saliva infiltration, S-saliva infiltration, and blank control from the 6, 24, 48, and 72 h exposure periods were assessed for their RNA levels. RNA was extracted from leaves using Trizol (Ambion Ltd. Co., Thermo Fisher Scientific, Waltham, MA, USA). The RNA solution (1 µL) was collected to determine the concentration and purity with an ultramicro ultraviolet spectrophotometer (NanoDrop 8000, Thermo Ltd. Co., East Lyme, CT, USA). First, strand cDNA was produced for the RNA samples of suitable purity using the reverse transcription kit ABM 5x All in One RT Mastermix (with accurate genomic DNA Removal Kit) (Qikai Ltd. Co., Guangzhou, China) and stored at −20 °C.

After aphid infestation and saliva infiltration, the relative expression levels of synthetic genes involved in the biosynthetic pathways for SA and JA were identified in the host plants by RT-qPCR. The target genes of the JA plant hormone production pathway were 13-lipoxygenase (*LOX*) and propylene oxide synthase (*AOS*). The target genes of the SA synthesis pathway were L-phenylalanine ammonia lyase (*PAL*) and isochorismate synthase (*ICS*). *Actin* was used as an internal reference gene. Primer Premier 5.0 software was used to design each primer. Specific primer information was detailed in Table 2. The principal synthesis genes associated with salicylic acid and jasmonic acid discussed in this article were all cited from the genomic study of *M. sativa* [35]. Corresponding primers were designed utilizing DNAman 9.0 software.

cDNA samples of alfalfa leaves were used as the template. TB Green^®^ Premix Ex TaqTM II (Baoshengwu Ltd. Co., Dalian, China) solution was used to perform the RT-qPCR. The biological specificity of each primer was evaluated by repeated biological treatments, and each primer contained three biological templates. The RT-qPCR reaction system (20 µL) consisted of: TB green premix ex Taq II 10.0 µL, upstream and downstream primers (10 pmol/µL) 0.5 µL each, cDNA template 2 µL, and sterilized water 7.0 µL. Test settings for the CFX96 Real-Time PCR Detection System (Bio-Rad, Ltd. Co., Waltham, MA, USA) were 95 °C for 30 s, 95 °C for 5 s, 60 °C for 35 s, and 40 cycles.

### 4.10. Statistical Analysis

The “R” software (R version 4.3.3) was used to examine the experimental data. Aphid mortality was tested for significant differences between the treatments using Log rank (Kaplan Cox) (The Survival and Survminer package of R software is used here). Then, the selections preferences of aphids for different treatments of alfalfa leaves were compared by Tukey’s honest significant difference (HSD) tests using a one-way analysis of variance (ANOVA) (*p* < 0.05) (The Car and Stats package of R software is used here). Two-factor variance under a generalized linear model was conducted to examine the effects of aphid strains and alfalfa cultivar on the concentration of jasmonic acid (normal distribution) and salicylic acid (normal distribution) in alfalfa leaves as well as the expression levels of key genes involved in jasmonic acid and salicylic acid synthesis (normal distribution) (*p* < 0.05) (The Car, Stats and Afex package of R software is used here). The multiple comparisons of various experimental indicators under different treatment combinations were tested using Tukey’s HSD method. For the data obtained from the RT-qPCR test, the 2^−ΔΔCT^ method was used to calculate the expression.

## 5. Conclusions

We reared two batches of *T. trifolii* on aphid-resistant alfalfa (Zhongmu No. 1) and aphid-susceptible alfalfa (WL343) for more than 50 generations to produce the resistant aphid strain (R-aphid) and susceptible aphid strain (S-aphid). The survival rates of the two aphid strains on Zhongmu No. 1 alfalfa were significantly different. Then, we extracted saliva from the R-aphids and S-aphids, denoted as R-saliva and S-saliva, respectively, and compared the effects on alfalfa leaves infested by S-aphids or infiltrated by S-saliva. After infestation by R-aphids or infiltration by R-saliva, the SA plant hormone signal pathway of alfalfa was overly activated. In contrast, the JA plant hormone signal pathway was inhibited, and the fitness of the aphids on alfalfa was improved. Therefore, we speculate that R-aphids could overstimulate the SA plant hormone signal pathway of the plant so as to inhibit the JA plant hormone signal pathway, thereby reducing the defensive ability of the plant and improving the survival of the aphids on alfalfa.

## Figures and Tables

**Table 1:** Six treatments in biological experiments of *T. trifolii* infected on two cultivars of alfalfa.

Order	*T. trifolii* Strain	Number of Aphids	Cultivar of Alfalfa	Repetition
1	R-aphid	30	Zhongmu No. 1	5
2	R-aphid	30	Zhongmu No. 1	5
3	No aphid	0	Zhongmu No. 1	5
4	S-aphid	30	WL343	5
5	S-aphid	30	WL343	5
6	No aphid	0	WL343	5

**Table 2:** Primers of RT-qPCR for genes involved in JA plant hormone and SA plant hormone defense response.

Gene Name	Primer Sequences (5′ to 3′)	Intention	Annealing Temperature (°C)
*AOS*-F	GTTGTTGTCCTCCTTGATGG	qPCR	53.0
*AOS*-R	GACGGCTACGTGATTTAAGG	qPCR	53.0
*LOX*-F	AACACATGGGTTCAGGACTA	qPCR	50.0
*LOX*-R	TTTACAGCTGCATGTAGAGC	qPCR	50.0
*ICS*-F	AGTTTCCTAAGCAAGCTCCT	qPCR	52.0
*ICS*-R	AAGCATTTCCACCTTCAACC	qPCR	52.0
*PAL*-F	GAAGGCTATCTTGCCAAAGG	qPCR	54.0
*PAL*-R	GGCACATAGCTGTGAACAAT	qPCR	54.0
*Actin*-F	CTGTACGGCAACATTGTTCT	qPCR	52.0
*Actin*-R	AGCAAGGATTGATCCTCCAA	qPCR	52.0

## Data Availability

All analyzed data are available in this paper.
